# Supplementary material for: Characterization of p38α autophosphorylation inhibitors that target the non-canonical activation pathway
Source: Nat Commun. 2023 Jun 12;14:3318. doi: 10.1038/s41467-023-39051-x (PMC10261013; doi:10.1038/s41467-023-39051-x)
Supplement: Supplementary file 1 — Supplementary Information [file 41467_2023_39051_MOESM1_ESM.pdf]

Supplementary information

Characterization of p38α autophosphorylation inhibitors that target the non-canonical activation pathway

Lorena González[1], Lucía Díaz[2], Joan Pous[1], Blazej Baginski[1], Anna Duran-Corbera[1], Margherita Scarpa[1], Isabelle Brun-Heath[1], Ana Igea[1], Pau Martin-Malpartida[1], Lidia Ruiz[1], Chiara Pallara[2], Mauricio Esguerra[2], Francesco Colizzi[1,6], Cristina Mayor-Ruiz[1], Ricardo M. Biondi[3], Robert Soliva[2], Maria J. Macias[1,4,7], Modesto Orozco[1,5,7], Angel R. Nebreda[1,4,7]

[1] Institute for Research in Biomedicine (IRB Barcelona), The Barcelona Institute of Science and Technology, 08028 Barcelona, Spain

[2] Nostrum Biodiscovery, 08034 Barcelona, Spain

[3] Instituto de Investigación en Biomedicina de Buenos Aires (IBioBA)-CONICET-Partner Institute of the Max Planck Society, Buenos Aires, Argentina

[4] ICREA, Pg. Lluís Companys 23, 08010 Barcelona, Spain

[5] Departament de Bioquímica i Biomedicina, Facultat de Biologia, Universitat de Barcelona, 08028 Barcelona, Spain.

[6] Present address: Department of Marine Biology and Oceanography, Institute of Marine Sciences ICM-CSIC, 08003 Barcelona, Spain

[7] These authors jointly supervised this work. emails: maria.macias@irbbarcelona.org, modesto.orozco@irbbarcelona.org, angel.nebreda@irbbarcelona.org

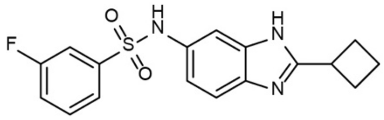

NC-27

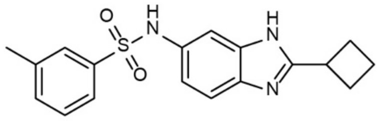

NC-39

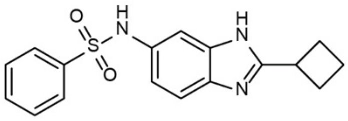

NC-37

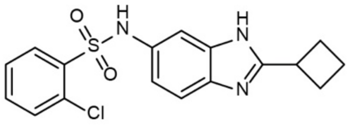

NC-42

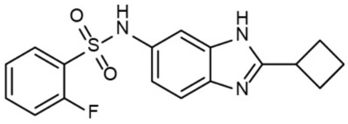

NC-38

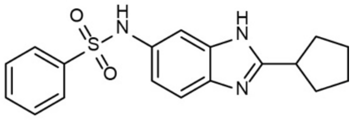

NC-60

Supplementary Fig. 1. Selected compounds that inhibit TAB1-induced p38α autophosphorylation more than 80%.

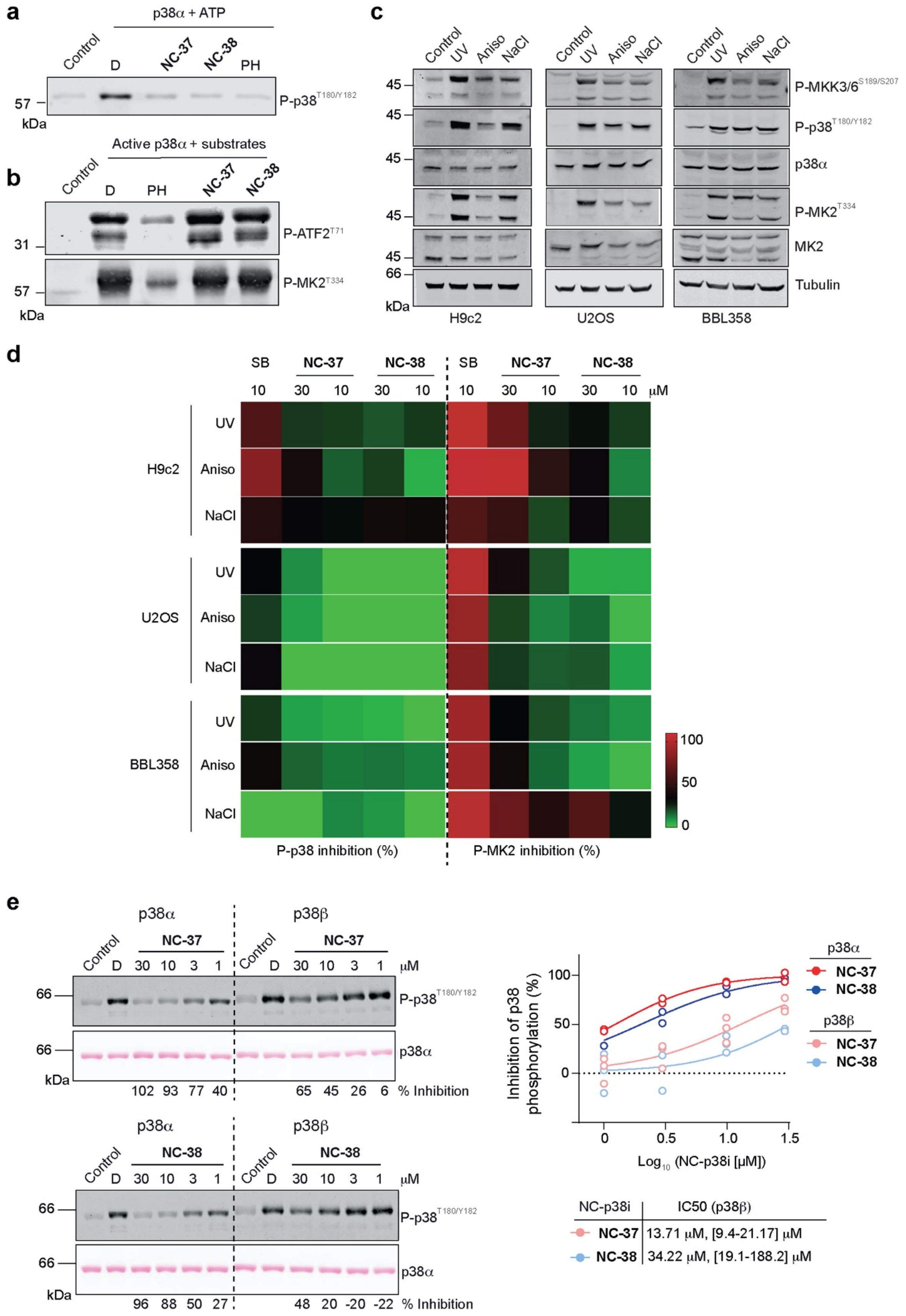

Supplementary Fig. 2. Effect of NC-p38i compounds on the stress-induced activation of the p38 pathway in cells, and on p38β autophosphorylation *in vitro*. a GST-p38α (0.5 μg)

was incubated with 150 μM ATP in 20 μl of autophosphorylation buffer, and in the presence of DMSO (D) or the compounds **NC-37** and **NC-38** (10 μM). Control, GST-p38α without ATP. After 2 h min at 37ºC, samples were analyzed by immunoblotting with antibodies anti-phospho-p38 (T180/Y182). Results are representative from n=2 experiments. **b** GST-p38α (0.5 μg) activated by MKK6 was incubated with the substrates GST-MK2 or GST-ATF2 (2 μg) in 20 μl of kinase buffer with ATP and in the presence of DMSO (D), PH797804 (PH, 2 μM) or the compounds **NC-37** and **NC-38** (10 μM). After 30 min at 30ºC, samples were analyzed by immunoblotting with the indicated antibodies. Controls, GST-MK2 and GST-ATF2 without active GST-p38α. Results are representative from n=2 experiments. **c** H9c2, U2OS and BBL358 cells were treated with UV irradiation (80 J/m2, 45 min), anisomycin (20 μM, 4 h), NaCl (200 mM, 4 h) or DMSO (Control), and total cell lysates were analyzed by immunoblotting with the indicated antibodies. Results are representative from n=3 experiments. **d** Heatmap showing the percentage of inhibition of p38α and MK2 phosphorylation in cells pre-treated with SB203580 (SB) or compounds **NC-37** and **NC-38** at the indicated concentrations for 2 h and then stimulated with UV, anisomycin or NaCl as in (**c**). Data represent the mean normalized to the DMSO-treated cells of n=3 experiments (except for H9c2 cells treated with anisomycin or NaCl, n=2). **e** Purified GST-p38α and GST-p38β proteins (1.5 μM, 2 μg in 20 μl) were incubated in autophosphorylation buffer with ATP and in the presence of DMSO (D) or the indicated concentrations of compounds **NC-37** and **NC-38**. Control, GST-p38α or GST-p38β without ATP. After 2 h at 37ºC, samples were analyzed by Ponceau staining and immunoblotting. The right panels show the inhibition curves of p38α and p38β autophosphorylation by different concentrations of compounds **NC-37** and **NC-38**. Results for GST-p38α, n=2 experiments, and for GST-p38β, n=3 experiments. Data were fitted using a nonlinear regression fit model (Graphpad Prism) to determine the IC50s for p38β autophosphorylation inhibition, with 95% confidence intervals indicated in brackets. Source data are provided as a Source Data file.

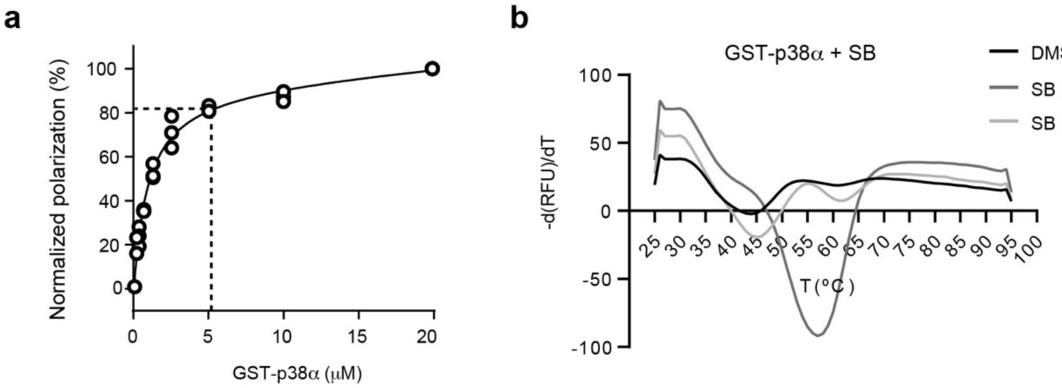

c

	μM	ΔTm (ºC)
SB	10	13 ± 0.5
	1	1.7 ± 0.9
**NC-37**	30	3.9 ± 0.5
	10	2.4 ± 0.5
	1	0.8 ± 0.6
**NC-38**	30	2.6 ± 0.5
	10	1.5 ± 0.3
	1	0.3 ± 0.2

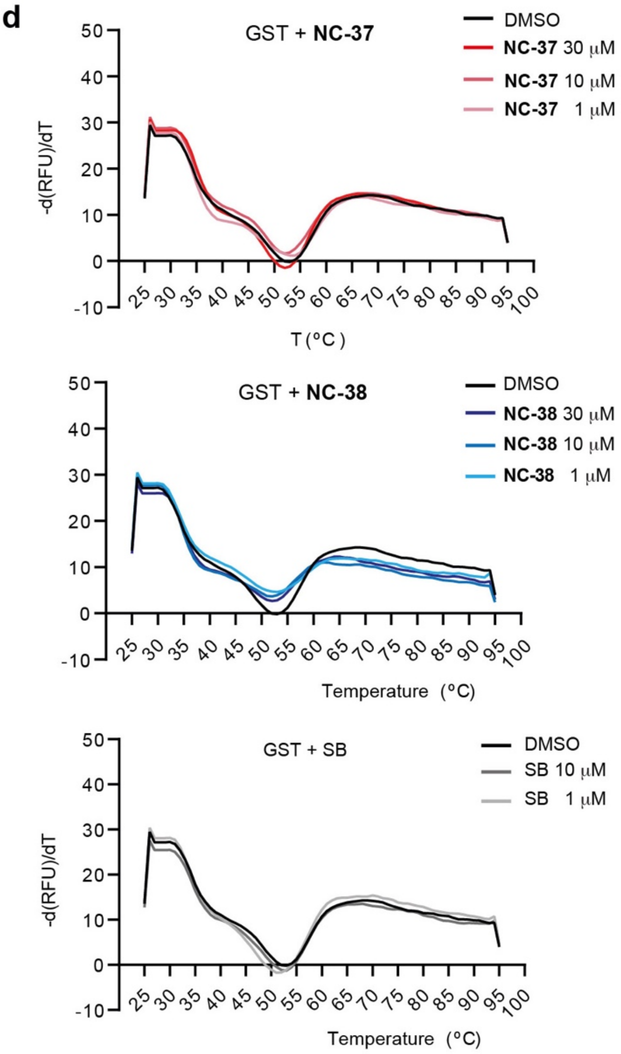

Supplementary Fig. 3. Effect of NC-p38i compounds on p38α denaturation as determined by fluorescence polarization analysis. a FITC-labelled TAB1$_{386\text{-}414}$ peptide (10 nM) was incubated with increasing concentrations of non-phosphorylated purified GST-p38α protein for 1 h, and binding was monitored using a fluorescence polarization (FP) assay (n=1 done in triplicates). Values were normalized as the percentage of FP signal, and data were fitted using a nonlinear regression fit single site-binding model (Graphpad Prism). **b**

Representative denaturation curves of non-phosphorylated GST-p38α in the presence of the indicated concentrations of SB203580 (SB). DMSO was used as control. **c** ΔTm values for GST-p38α in the presence of SB or compounds **NC-37** or **NC-38** at the indicated concentrations (ΔTm = Tm inhibitor - Tm DMSO). SB was used as a positive control for p38α stabilization. Results are shown as mean ± SD of n=3 biologically independent experiments. **d** Representative denaturation curves of purified GST protein in the presence of SB and compounds **NC-37** or **NC-38** at the indicated concentrations. DMSO was used as control. Source data are provided as a Source Data file.

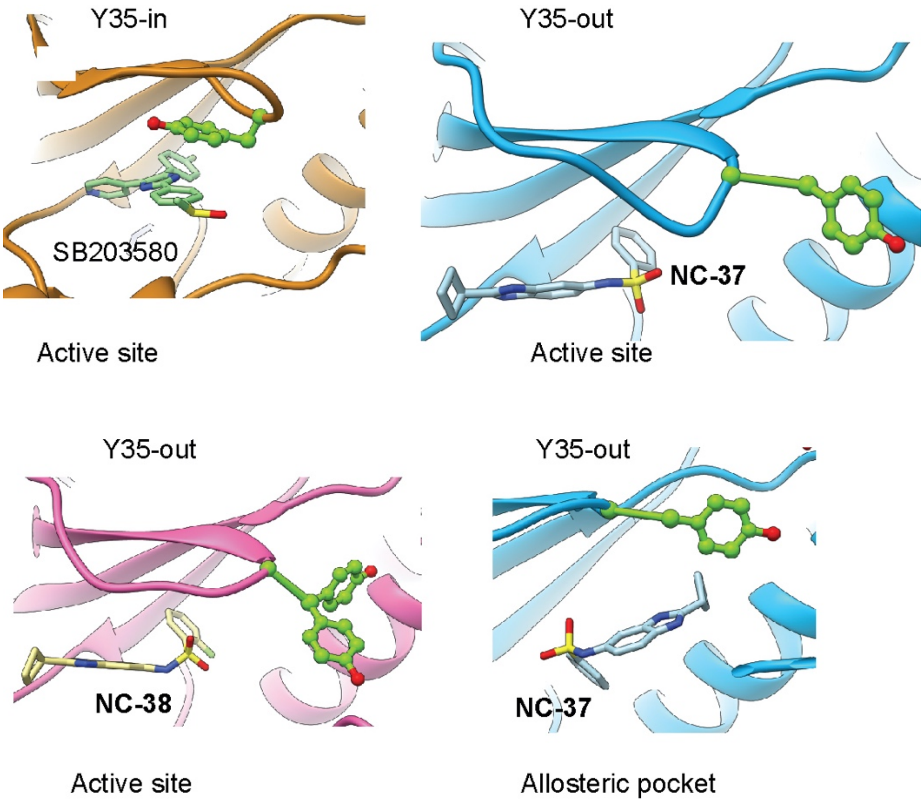

Supplementary Fig. 4. Orientation of the Tyr35 side chain in p38α bound to NC-p38i compounds. The panels show the Y35-in (1A9U structure, in orange) and the Y35-out (both 7Z9T structure in cyan and 7PVU structures in magenta). Compounds are labelled in the figure for clarity.

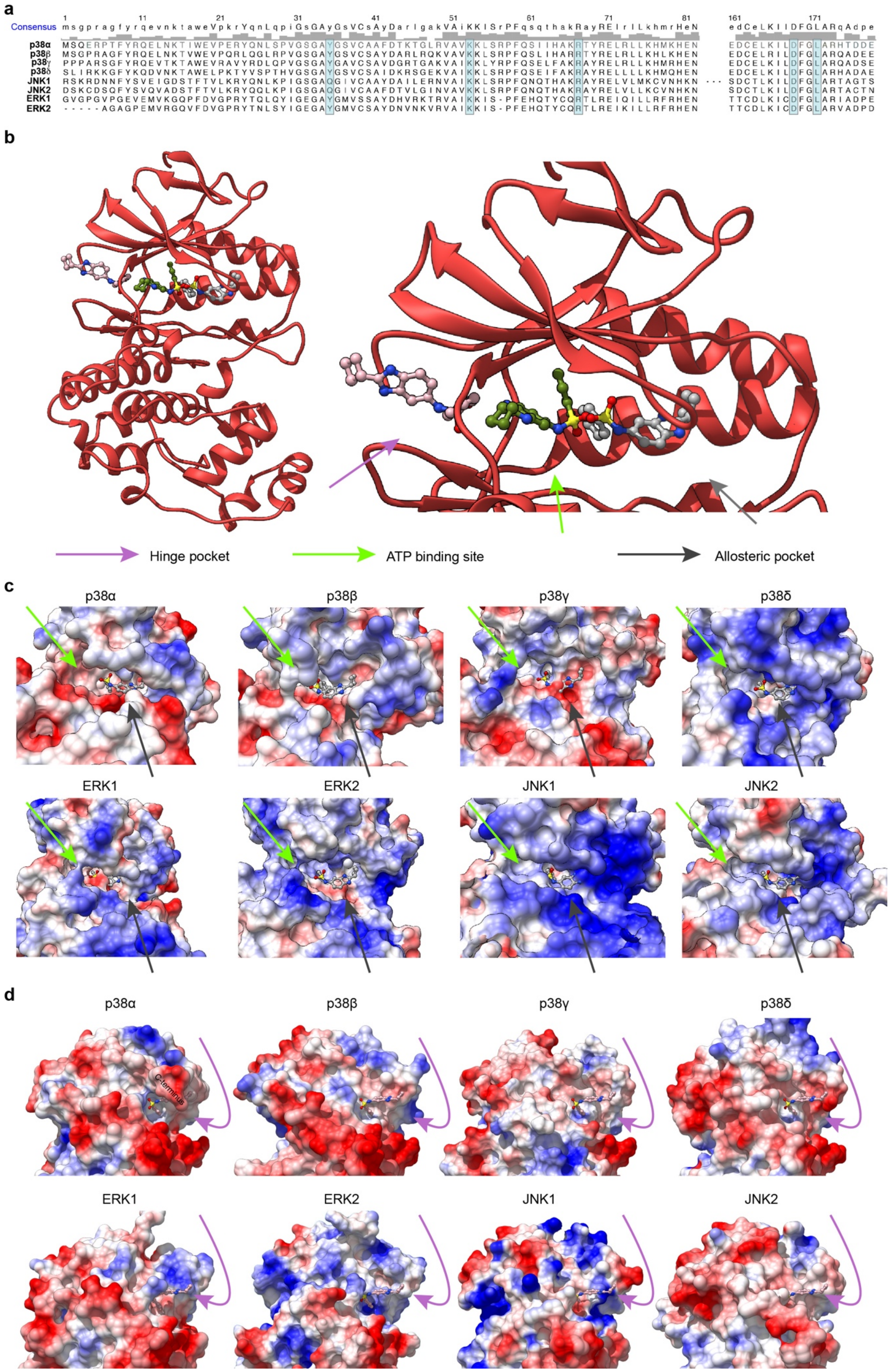
a
Consensus
p38α
p38β
p38γ
p38δ
JNK1
JNK2
ERK1
ERK2
b
Hinge pocket
ATP binding site
Allosteric pocket
c
p38α
p38β
p38γ
p38δ
ERK1
ERK2
JNK1
JNK2
d
p38α
p38β
p38γ
p38δ
C-terminus
ERK1
ERK2
JNK1
JNK2

Supplementary Fig. 5. Comparison of the ATP binding site in different MAPKs. a Sequence alignment of several MAPKs with the essential residues involved in binding to ATP highlighted in blue. **b** The three binding sites of NC-p38i compounds observed in p38α (the hinge pocket, the ATP binding site and the allosteric pocket) are indicated with arrows. **c** and **d** Charge distribution at the ATP binding site and the allosteric pocket (**c**), and at the hinge pocket (**d**) of the indicated MAPKs. Molecules bound at the allosteric and hinge pockets are shown.

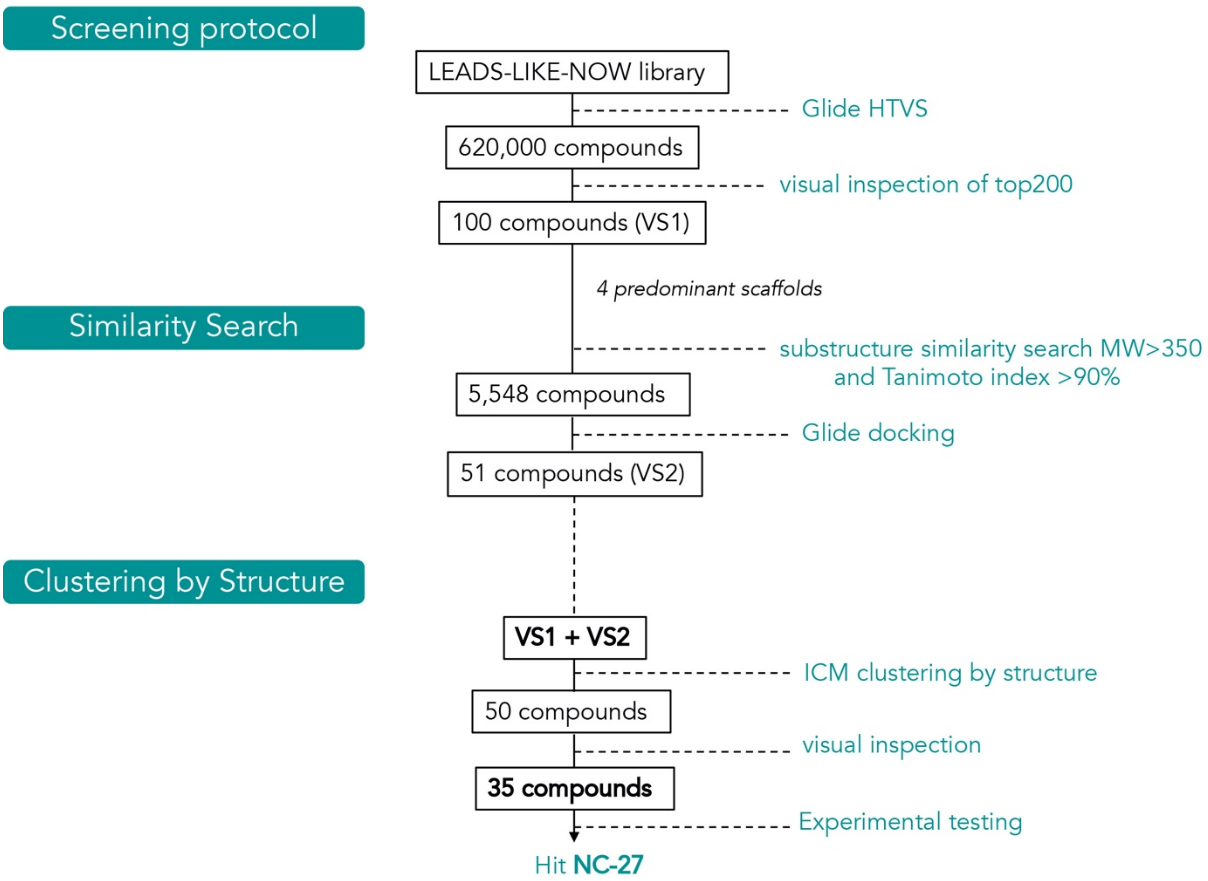

Supplementary Fig. 6. Flowchart of the screening campaign. Workflow representation of the Screening protocol performed using the LEADS-LIKE-NOW library from ZINC and subsequent Similarity Search using virtual hit compounds as queries. The number of molecules is specified at each stage as well as the identification of the hit compound **NC-27.** HTVS, High-Throughput Virtual Screening. VS, Virtual Screening.

a

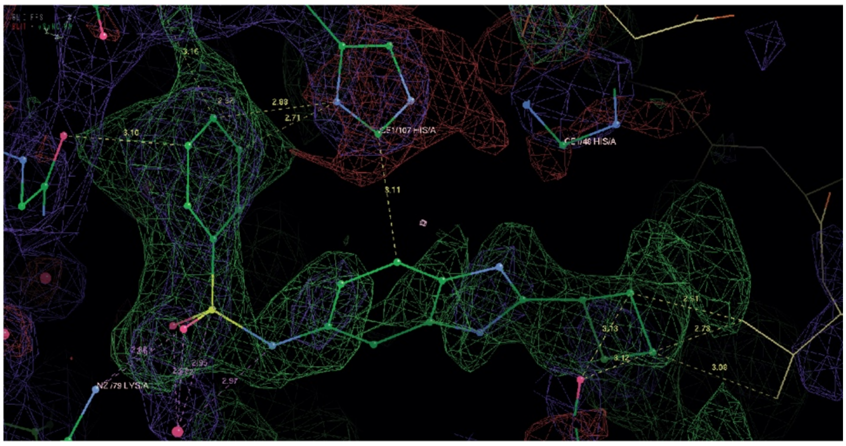

CC(1,2): 0.4675; CC(1,3):0.5741; CC(2,3): 0.3623

b

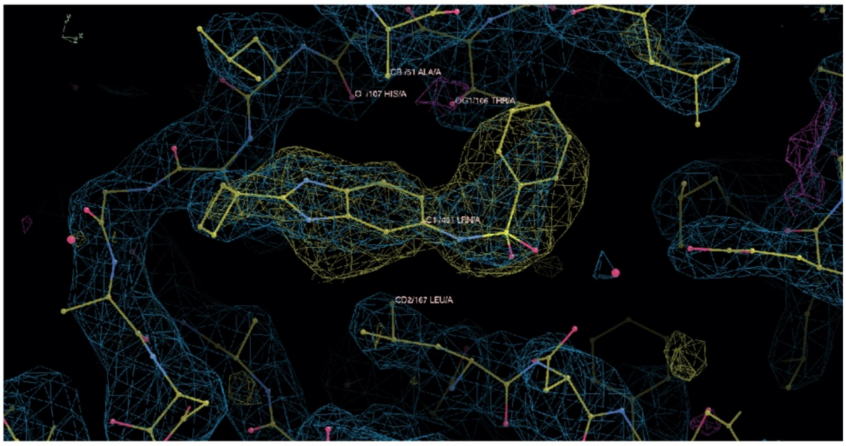

CC(1,2): 0.6654; CC(1,3):0.8817; CC(2,3): 0.6079

c

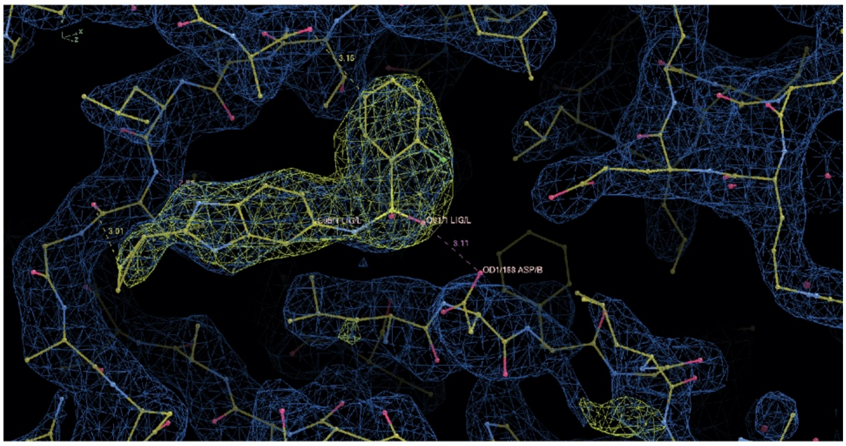

CC(1,2): 0.5840; CC(1,3):0.8810; CC(2,3): 0.6106

Supplementary Fig. 7. Polder and electron density maps for the NC-p38i compounds and surrounding residues of p38α. Polder (in green/red) and electron density maps (2Fc-Fo, in blue) are indicated. CC values are indicated below each panel. **a** Compound **NC-37** in 7Z6I. Polder at ±2 σ , 2Fc-Fo map at 1 σ. **b** Compound **NC-37** in 7Z9T, chain A. Polder at ±3 σ , 2Fc-Fo map at 1 σ. **c** Compound **NC-38** in 7PVU, chain A. Polder at ±3 σ , 2Fc-Fo map at 1 σ.

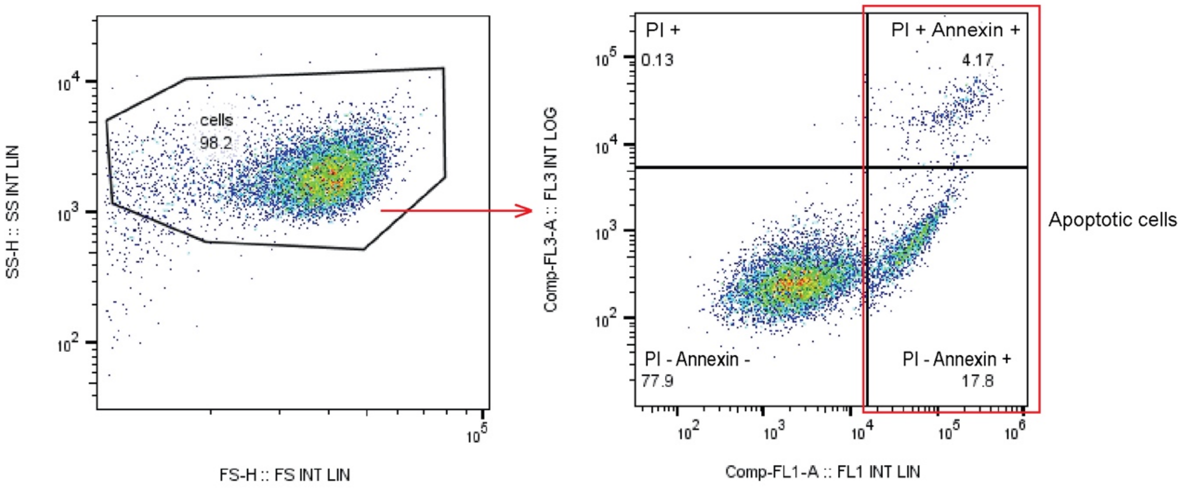

Supplementary Fig. 8. FACS gating strategy used to analyze SIR-induced cell death in H9c2 cells.

Supplementary Table 1. Commercial compounds used in p38α autophosphorylation experiments.

Compound	Supplier	Reference
**NC-1**	Toronto Research Chemicals	S116500
**NC-8**	Vitas M Lab	STK576573
**NC-19**	SPECS	AH-487/41708816
**NC-26**	Enamine	Z1386755546
**NC-27**	Enamine	Z1359501456
**NC-33**	Enamine	Z1139358721
**NC-37**	Enamine	Z1359501710
**NC-38**	Enamine	Z1359501532
**NC-39**	Enamine	Z1411273651
**NC-42**	Enamine	Z1359501546
**NC-60**	Otava LTd	4616224

Supplementary Table 2. Plasmids used for recombinant protein expression.

**Recombinant DNA**		
**Protein**	**Vector**	**Resistance**
GST-p38α (Hu)	pGEX-4T-3 and pGEX-6P-1	Ampicillin
GST-p38β (Hu)	pGEX-4T-1	Ampicillin
MBP-MKK6^{DD} (Hu)	pMalc2 [1]	Ampicillin
GST-MK2 (46-400) (Hu)	pGEX-KG [2]	Ampicillin
GST-ATF2 (19-96) (Hu)	pGEX-KG [3]	Ampicillin
His-SUMO-p38α C162S (Ms)	pOPINS	Kanamycin
GST	pGEX-6P-1	Ampicillin

Supplementary Table 3. Antibodies used for immunoblotting.

Primary antibodies			
**Antibody**	**Dilution**	**Company**	**Reference**
Caspase-3	1:1000	Cell Signaling	9662
GFP (B-2)	1:1000	Santa Cruz	sc9996
ERK1/2 (3A7)	1:1000	Cell Signaling	9107
MK2	1:1000	Cell Signaling	3042
MK2 phospho-T334 (27B7)	1:500	Cell Signaling	3007
ATF2 phospho-T71	1:1000	Cell Signaling	9221
p38α	1:1000	Cell Signaling	9218
p38 phospho-T180/Y182	1:1000	Cell Signaling	9211
p38 phospho-T180/Y182 (clone 36/p38)	1:1000	BD Biosciences	612288
MKK3/6 phospho-S189/S207	1:1000	Cell Signaling	9231
TAB1	1:1000	MRC, Dundee (UK)	S823A
Tubulin (DM1A)	1:10000	Sigma	T9026
**Secondary antibodies**			
Goat anti-Rabbit IgG (AlexaFluor 680)	1:5000	Invitrogen	A21076
Donkey anti-Mouse IgG (IRDye 800CW)	1:5000	Licor	926-32212
Donkey anti-Sheep IgG (HRP)	1:5000	Santa Cruz	sc2473
Donkey anti-Goat IgG (IRDye 800CW)	1:5000	Licor	926-32214

Supplementary Table 4. Crystallization, data collection and refinement statistics.

PDB ID	7Z6I	7Z9T	7PVU
Contents	p38α-C162S + SB203580 + **NC-37**	p38α-C162S + **NC-37** + ATPγS	p38α-C162S + **NC-38**
**Crystallization**			
Conditions	0.087 M Ca Acetate, 0.1M MES pH 6, 7.1% PEG 550MME	27.5% PEG 3350, 0.1M BIS-TRIS pH 6.8	27.0% PEG 3350, 0.1M BIS-TRIS pH 6.8
Temperature	20°C	20°C	20°C
Cryo protection	50% glycerol + 25% PEG 3350	No	No
**Data collection**			
Beamline data	Xaloc BL13 (Alba, Barcelona) Detector: Pilatus 6M, Wavelength: 0.9792 Å, Temperature:100K		
Distance	392.16 mm	508.10 mm	392.22 mm
**Data processing**			
Mosaicity	0.32°	0.29°	0.35°
Space group	$P\ 2_1\ 2_1\ 2_1$	$P\ 2_1$	$P\ 2_1$
Cell	65.14, 74.57, 77.55 Å	67.14, 68.07, 79.98Å β=94.78°	66.92, 67.97, 79.69Å β=94.75°
Resolution range	77.55 - 2.25 Å (2.37 – 2.25 Å)	79.70 - 2.60 Å (2.74 - 2.60 Å)	77.69 – 1,82 Å (1.92 – 1.82 Å)
Multiplicity	5.6 (5.6)	3.0 (3.1)	5.8 (5.8)
Completeness	100% (100%)	99.2 % (99.0%)	100% (100%)
I/**σ**(I)	16.5 (9.2)	9.2 (3.5)	16.0 (4.4)
Rmerge	0.092 (0.451)	0.086 (0.465)	0.057 (0.453)
Wilson B factor	29 $Å^2$	44 $Å^2$	41 $Å^2$
**Structure solution and refinement**			
Method	Molecular Replacement (4LOO)		
R factor	0.19 (0.21)	0.21 (0.27)	0.21 (0.30)
Free R factor	0.25 (0.27)	0.26 (0.32)	0.25 (0.32)
RMS bonds	0.0032 Å	0.0038 Å	0.0100 Å
RMS angles	1.18°	1.39°	1.18°
RMS chiral	0.05 $Å^3$	0.05 $Å^3$	0.08$Å^3$
<B>	36 $Å^2$	60 $Å^2$	70 $Å^2$
Ramachandran Favored, Outliers	93.0%, 2.3%	86.0%, 6.6%	92.9%, 1,8%

Highest resolution shells are indicated in parentheses. PEG 3350 in w/v

Supplementary References

1. Alonso, G., Ambrosino, C., Jones, M. & Nebreda, A.R. Differential activation of p38 mitogen-activated protein kinase isoforms depending on signal strength. *J Biol Chem* **275**, 40641-8 (2000).
2. Ben-Levy, R. et al. Identification of novel phosphorylation sites required for activation of MAPKAP kinase-2. *EMBO J* **14**, 5920-30 (1995).
3. Livingstone, C., Patel, G. & Jones, N. ATF-2 contains a phosphorylation-dependent transcriptional activation domain. *EMBO J* **14**, 1785-97 (1995).
